# Putative Calcium Channels CchA and MidA Play the Important Roles in Conidiation, Hyphal Polarity and Cell Wall Components in *Aspergillus nidulans*

**DOI:** 10.1371/journal.pone.0046564

**Published:** 2012-10-12

**Authors:** Sha Wang, Jinling Cao, Xiao Liu, Hongqin Hu, Jie Shi, Shizhu Zhang, Nancy P. Keller, Ling Lu

**Affiliations:** 1 Jiangsu Key Laboratory for Microbes and Functional Genomics, Jiangsu Engineering and Technology Research Center for Microbiology, College of Life Sciences; Nanjing Normal University, Nanjing, China; 2 Department of Plant Pathology, Department of Bacteriology and Department of Medical Microbiology and Immunology, UW-Madison, Madison, Wisconsin, United States of America; Universidade de Sao Paulo, Brazil

## Abstract

Although the high affinity Ca^2+^ channel, Cch1, and its subunit Mid1 have been investigated and evaluated in yeast and some of filamentous fungi, little is known about the function of their homologs in the *Aspergilli*. Here, we have functionally characterized the yeast homologs, CchA and MidA, in *Aspergillus nidulans* using conditional and null deletion mutants. CchA and MidA not only have functional benefits of fast growth, which is consistent with Cch1 and Mid1 in yeast, but also have unique and complex roles in regulating conidiation, hyphal polarity and cell wall components in low-calcium environments. The defect of CchA or MidA resulted in a sharp reduction in the number of conidiospores, accompanied by abnormal metulae, and undeveloped-phialides at a higher density of inoculum. Most interestingly, these conidiation defects in mutants can, remarkably, be rescued either by extra-cellular Ca^2+^ in a calcineurin-dependent way or by osmotic stress in a calcineurin-independent way. Moreover, the fact that the phenotypic defects are not exacerbated by the presence of the double deletion, together with the Y2H assay, indicates that CchA and MidA may form a complex to function together. Our findings suggest that the high-affinity Ca^2+^ channel may represent a viable and completely unexplored avenue to reduce conidiation in the *Aspergilli*.

## Introduction

Calcium-mediated signaling mechanisms are widely employed in eukaryotes and are implicated in the regulation of diverse biological processes including gene expression, exocytosis, cytoskeletal rearrangement, and cell morphology [1], [2], [3]. Intracellular calcium ion (Ca^2+^) concentrations change in response to environmental stimuli and physiological signals, which are followed by organismal adaptation [4], [5], [6]. In baker's yeast, *Saccharomyces cerevisiae* (*S. cerevisiae*), at least two different carrier systems have been identified—a high-affinity calcium influx system (HACS) and a low-affinity calcium influx system (LACS) [7], [8]. The HACS consists of at least two known subunits, Cch1 and Mid1, which act as the major calcium entry route when calcium availability is low [9]. Cch1 is a homolog to the catalytic α-subunits in mammals whereas Mid1 is possibly a regulatory subunit without homology to any known human β subunits [7], [8], [10]. To date, the predicted homologs of Cch1 and Mid1 have been identified in many fungi including ascomycetes and basidiomycota fungi [11]. In addition to *S. cerevisiae*, characterization of Cch1 or Mid1 has been undertaken in a variety of fungi such as the saprophytes *Schizosaccharomyces pombe* [12] and *Neurospora crassa* [13]; animal pathogenic fungi *Candida albicans* [4], and *Cryptococcus neoformans* [14], and plant pathogenic fungi *Gibberella zeae* [15], [16], *Claviceps purpurea* [17] and *Uromyces appendiculatus* [18]. In all of these fungi, Cch1 and Mid1 mutants consistently cause significant reduction of calcium uptake [11]. In yeast, loss of Mid1 or Cch1 results in cell death upon exposure to α-factor in calcium-limited medium. Therefore, the name *mid1* (*m*ating-*i*nduced *d*eath) was initially used to describe this gene [11], [19]. However, in the filamentous fungus *N. crassa*, the *mid1* mutant mates successfully, indicating the role of Mid1 protein differs from that of the homologous gene product in yeast [13]. Deletion of *mid1* gene has no observable effect on *N. crassa* sporulation processes but deletion of *mid1* in *G. zeae* affects ascospore discharge [13], [15]. These predicted calcium channel proteins are also important in virulence in pathogenic fungi. In the phytopathogenic fungus *C. purpurea*, deletion of *mid1* results in complete loss of virulence in infected rye plants [17]. Similarly, mice infected with the Cch1 mutant in pathogenic yeast *C. neoformans* have improved survival rates [14]. Notably, recent studies have indicated that, in the human fungal pathogens *C. albicans* and *C. neoformans*, HACS is fundamental for sensing and adaptation to the human host milieu; additionally, these studies have shown that the activities of HACS were required in response to various stresses and tolerance of antifungal compounds [14], [20], [21]. These latter results suggest that Cch1 and Mid1 might be suitable candidates as novel drug targets. Thus, further studies are necessary for better understanding the complexity of Cch1 and Mid1 in different fungi. One important genus with no reports of Cch1 or Mid1 function is *Aspergillus*. This genus contains species with tremendous impact on public health: both positively and negatively [22]. *Aspergillus nidulans* (*A.nidulans*) is considered a premier model fungus for filamentous fungi especially for other *Aspergilli* due to its well-established genetic system [23], [24]. Similar to the pathogenic *Aspergilli*, *A. nidulans* produces small, hydrophobic conidia that disperse easily into the air and can survive a broad range of environmental conditions [23], [25]. Therefore, conidiation is a possible target for controlling propagation or dispersal in the *Aspergilli* [26], [27], [28]. In this study, we focus on the physiological and genetic functions of CchA and MidA, homologs of Cch1 and Mid1, in *A. nidulans*. Our results suggest that both CchA and MidA are critical for conidial development, hyphal polarity establishment, and cell wall components in low-calcium environmental conditions.

## Materials and Methods

### Strains, media, culture conditions, and transformation

A list of *A. nidulans* strains used in this study is given in Table 1. TN02A7, a deletion strain of a gene required for non-homologous end joining in double-strand break repair [29], was used in all transformation experiments. The following media [30], [31] were used: YAG, 2% glucose, 0.5% yeast extract, trace elements as needed; YUU, YAG supplemented with 5 mM uridine and 10 mM uracil; YAGK, YAG with 0.6 M KCl; YUUK, YUU with 0.6 M KCl; MMPDR, minimal medium with 2% glucose, nitrate salts, trace elements, 0.5 mg L^−1^ pyridoxine, 2.5 mg L^−1^ riboflavin, pH 6.5, trace elements and nitrate salts were added to the media as described previously [30]; MMPGR, same as MMPDR but replacing 2% glucose with 1% glycerol (v/v). For solid media, 2% agar was added. Growth conditions, crosses and induction conditions for *alc*A(*p*)-driven expression were as previously described [32]. Transformation was done according to a method described [33], [34].

**Table 1:** *A. nidulans* strains used in this study.

Strain	Genotype	Reference or source
TN02A7	*pyrG89*; *riboB2*; *nkuA*::*argB2*; *pyroA4*; *veA1*	[29]
WJA01	*riboB2*; *nkuA*::*argB2*; *pyroA4*; *veA1*	This study
CJA08	*pyrG89*; *riboB2*; *nkuA*::*argB2*, Δ*midA*::*pyrG*; *pyroA4*; *veA1*	This study
CJA09	*pyrG89*; *riboB2; nkuA*::*argB2*, Δ*midA(*Δ*C)*::*pyrG*; *pyroA4*; *veA1*	This study
WSA05	*pyrG89*; *riboB2*; *nkuA*::*argB2*; *pyroA4;* Δ*cchA*::*pyrG*; *veA1*	This study
WSA06	*pyrG89*; *riboB2*; *nkuA*::*argB2*, Δ*midA*::*pyroA; pyroA4;* Δ*cchA*::*pyrG*; *veA1*	This study
HHA01	*pyrG89*; *riboB2*; *nkuA*::*argB2*, *alcA(p*)::CFP*-*MidA::*pyr-4*; *pyroA4*; *veA1*	[5]
HHA02	*pyrG89*; *riboB2*; *nkuA*::*argB2*; *pyroA4*; *alcA(p)*::YFP*-*CchA::*pyr-4*; *veA1*	[5]

### BLAST, alignments and topology analysis

BLASTp searches of the genomes of *H. sapiens*, *M. musculus*, *D. melanogaster*, *A. nidulans*, *A. fumigatus*, *A. flavus*, *A. clavatus*, *A. terreus*, *A. oryzae*, *A. niger*, *P. chrysogenum*, *N. crassa*, *P. marneffei*, *C. posadasii*, *G. zeae*, *C. purpurea*, *C. neoformans*, *C. albicans*, *S. cerevisiae* were carried out against the National Center for Biotechnology (NCBI) genomic protein databases. Multiple sequence alignments and homology distance were made using Clustal W2.1.

For the results of predicted topology of CchA or MidA: (1) The presence of multiple transmembrane domains was confirmed using the SMART database (http://smart.embl-heidelberg.de/) and the TMpred program (http://ch.embnet.org/software/TMPRED_form.html); (2) The EF-hand region of CchA was predicted by the Central Aspergillus REsource (CADRE) database (http://www.cadre-genomes.org.uk/Aspergillus_nidulans/Info/Index); (3) The presence of conserved domains was confirmed using the conserved domains database (NCBI) (http://www.ncbi.nlm.nih.gov/Structure/cdd/wrpsb.cgi); (4) The hydropathy analysis was performed by Kyte-Doolittle hydropathy program to predict the putative membrane spanning segments and pore loop portions of the putative channel; (5) The 12 putative N-glycosylation sites of MidA are identified by the NetNGlyc 1.0 server (http://www.cbs.dtu.dk/services/NetNGly).

### Tagging of CchA with YFP and MidA with CFP *in vivo*

To generate the *alcA*(*p*)::YFP-*cchA* vector, a 1000 bp *cchA* fragment was amplified from the genomic DNA in the wild-type strain TN02A7 with primers *cchA*-5′ (TTATGCGGCCGCTGGCGTCAAATAGCCATGAC; NotI site underline) and *cchA*-3′ (TTGGTCTAGACCATTCCGATTGCGCTGATTC; XbaI site underlined) (Table S1) and then ligated into the plasmid vector pLB-nudF [35] in which nudF was cut off by NotI and XbaI yielding plasmid pLB*^alcA(p)^* ^::YFP-*cchA*^ which contains YFP-N under the regulation of the *alc*A promoter and *N. crassa pyr*4 as marker. To create the *alcA*(*p*)::CFP-*midA* vector, a 610 bp *midA* fragment amplified from the wild-type genome with primers mid-5′ (ACTTGGCGGCCGCTGCAACGGCAAAAC; NotI site underline) and mid-3′ (TACACCCGGGTACACCTCAGATGTAG; SmaI site underline) (Table S1) was ligated with the plasmid vector pLB-γ-Tubulin [35] in which γ-Tubulin was cut off by NotI and SmaI. The resulting plasmids pLB*^alcA(p)^* ^::YFP-*cchA*^ and pLB*^alcA(p)^* ^::CFP-*midA*^ were then transformed into the TN02A7 strain. The homologous recombination was confirmed by diagnostic PCR and Western blotting analysis, generating the *alcA*(*p*)::YFP-*cchA* conditional strain, referred as HHA02 and the *alcA*(*p*)::CFP-*midA* strain HHA01 [5].

### Constructions of the *cchA* and *midA* deletion strains

Deletion mutants were created by double joint PCR [36]. The *Aspergillus fumigatus pyr*G gene from plasmid pXDRFP4 was used as a selectable nutritional marker for transformation. The information of primers for fusion PCR products are listed in Table S1. The full deletion cassettes for *cchA* (970 bp *cchA* up-stream, 1.9 kb *pyr*G, 1000 bp *cchA* down-stream) and for *midA* (566 bp *midA* up-stream, 1.9 kb *pyr*G and 541 bp *midA* down-stream) obtained by recombinant PCR using the primer pairs p1–p3, p4–p6 and nested primers p2–p5 respectively (Table S1), were purified and used to transform into TN02A7. The homologous recombination was confirmed by Southern blotting analysis, generating the Δ*cchA* strain WSA05 and *ΔmidA* strain CJA08. To construct *cchA* and *midA* double deletions, *midA* gene was replaced by *pyro*A insertion from plasmid pQa-pyroA as a selectable nutritional marker in *cchA* deletion background. The transformants were selected on MM media without pyridoxine. The homologous recombination was confirmed by diagnostic PCR, generating the double deletion Δ*cchA*/Δ*midA* strain WSA06. A similar strategy was used to construct the truncated *midA* strain by using primers L-flank-5′ (truncated) and L-flank-3′(truncated) for the up-stream region; mid-p4 and mid-p6 for the down-stream region, nested primers mid-p2(truncated) and mid-p5 for the fusion product, and the resultant linear fragment was purified and used to transform TN02A7.

### Immunoblotting experiments and Southern hybridization

To extract proteins from *A. nidulans* mycelia, conidial spores from *alcA*(*p*)::YFP-*cchA*, *alcA*(*p*)::CFP-*midA* and the wild-type strains were inoculated in MMPGR liquid medium, then shaken at 220 r.p.m. on a rotary shaker at 37°C for 20 h. Total proteins were extracted and separated on a 10% tris-glycine SDS gel. Immunoblotting experiments were done as previously described [37] with anti-GFP (N-terminal) mouse monoclonal primary antibodies (Roche Applied Science). For Southern hybridization, Zeta-Probe membranes (Bio-Rad) were probed with DIG-labeled probes, and processed as described in the manufacturer's protocol (Roche Applied Science).

### Scanning electron microscopy

Scanning electron microscopy examination was carried out mainly as described previously [38], [39], [40]. In brief, for sample preparation, sporulating colonies that had grown on MMPDR for 2 d at 37°C were fixed firstly for 2 h in 4% glutaraldehyde with 0.01 M phosphate buffer (pH 7.0) at room temperature, then washed by 0.01 M phosphate buffer (pH 7.0). The samples were then dehydrated with a graded ethanol series of 30, 50, 70, 80, 90, and 100% ethanol, 10 min per step. The samples were then treated with fresh 100% ethanol for 30 min. The ethanol was replaced with isoamyl acetate for 30 min, critical point dried (Polaron E3000, Series II), sputter coated with gold and observed by scanning electron microscopy (JSM-5610LV, VANTAGE).

### Plate assays

Unless indicated elsewhere, MMPDR in which pH was adjusted to 6.5 was used as plate assays. The supplements were added into media prior to autoclaving. For each test at least three plates were prepared for each strain. To assess the role of elevated Ca^2+^ in the medium, MM was supplemented with 5, 10, 20, 40 mM CaCl_2_, respectively. The influence of osmotic stress or ionic stress was tested by adding 800 mM NaCl, 600 mM KCl, 1 M Sorbitol, 1 M Sucrose, or 200 mM LiCl into MMPDR, respectively. For inoculum-size-dependent test, aliquots of 2 µl from a series of 10-fold dilutions derived from a starting suspension of 10^9^ conidia ml^−1^ of the indicated strains were spotted onto MMPDR, and then all plates were incubated at 37°C for 48 h. For chemical sensitive experiments, 1 µM FK506 (Cat No. F4679 from Sigma Co.), 40 µg ml^−1^ Calcofluor White and 400 µg ml^−1^ Congo Red were added to media after sterile filtration as indicated in the text. After cultured for 2 days at 37°C, the colonies were observed and imaged unless stated otherwise.

### Cell wall analysis

Sensitivity to cell wall disrupting agents was determined by inoculating conidia on MMPDR amended by 40 µg ml^−1^ Calcofluor White or 400 µg ml^−1^ Congo Red respectively. All incubations were at 37°C for two days. The visualization of chitin was performed by staining with Calcofluor White as previously described [41]. Chitin assays for cell wall were performed using the amount of N-acetyl-glucosamine present in the alkali-insoluble cellular fraction. Briefly, 3×10^6^ conidia ml^−1^ from each strain were grown for 24 h at 37°C with shaking at 220 rpm in flasks containing YAG liquid medium, and then hyphae were harvested by vacuum filtration. 5 mg of lyophilized hyphal powder was re-suspended in 3 ml of saturated KOH and incubated at 130°C for 1 h. After centrifugation at 20, 000 g for 5 min, the supernatant was used to quantify chitin using the protocol has been described previously [41], [42]. The mean chitin content was determined from the analysis of three independent supernatants. For quantification of β-1, 3-glucan levels between wild-type and mutants, β-1, 3-glucan content was examined using the aniline blue assay using the protocol described previously [41] since aniline blue is highly specific for β-1, 3-glucan which is composed largely or entirely of β-1, 3-glucosidic linkages. Briefly, twenty milligrams of lyophilized hyphal powder from each strain was re-suspended in 1 ml of 1 M NaOH, and then sonicated for 30 s, followed by incubation at 52°C for 30 min. After centrifugation at 6000 rpm for 5 min, 250 µl supernatant of each sample was added with a volume of 925 µl of aniline blue mix (0.067% aniline blue, 0.35 M HCl, 0.98 M glycine-NaOH, pH 9.5) in a new tube, incubated for an additional 30 min at 52°C then allowed to cool at room temperature for 30 min. Lastly, fluorescence readings were acquired on a SPECTRAmax M2 fluorimeter (Molecular Devices, Sunnyvale, CA) at 405 nm excitation and 460 nm emission with 200 µl above mixed liquor in the 96-well plate. Values are expressed as the percent change in relative fluorescence units per milligram of mycelial tissue, using wild type as a control. Final results represent the averages from three independent experiments. For β-1, 3-glucan analysis by flow cytometry, the cell wall β-1, 3-glucan was labeled using an anti-β-1, 3-glucan antibody (Biosupplies, Parkville, Australia) as previous described with some modifications [43], [44]. Briefly, fungal conidia were harvested by centrifugation, rinsed with distilled water, and then fixed with 4% (v/v) para-formaldehyde for 30 min, washed four times using phosphate buffered saline buffer (0.01 M PBS, 137 mM NaCl, 2.7 mM KCl, 8.1 mM Na_2_HPO4, 1.5 mM KH_2_PO4, pH 7.4) before being suspended in 1% (v/v) Tween-20 in PBS buffer. The samples were then incubated with the monoclonal β-1, 3-glucan-specific antibody (Biosupplies, Parkville, Australia) (0.1 mg ml^−1^ in PBS buffer) as the primary antibody for at least 3 h at room temperature before incubation with Alexa Fluor 546 goat anti-mouse IgG antibody (Invitrogen) (0.1 mg ml^−1^ in PBS buffer) as the secondary antibody for at least 2 h in the dark. Fluorescent signal was quantified using a Becton Dickinson FACSort (Fluorescence activated cell sorter), excitation wavelength 488 nm, emitted light detector 546 nm, adjusted to a fixed channel using standard Brite Beads, Coulter, USA) prior to determining fluorescence. Data acquisition and manipulation was performed with Cell Quest and FACSExpress v3 and fluorescence was measured for 20,000 conidia.

### Fluorescence staining and microscopic observations

For microscopic observations, conidia were inoculated onto pre-cleaned glass coverslips overlaid with liquid media. Strains were grown on the coverslips at 37°C for the time as indicated prior to observation under microscope. The visualization of chitin was performed by staining with Calcofluor White as previously described (Sigma Aldrich, St. Louis). For the staining of Spitzenkörper, a membrane-selective fluorescent vital dye FM4-64 was used as previously described [45]. Briefly, the samples were loaded by 5 µM FM4-64 at 25°C for 4–15 min within MMPDR, and then washed out the dye by using fresh medium without FM4-64. Differential interference contrast (DIC) and fluorescent images of the cells were collected with a Zeiss Axio imager A1 microscope (Zeiss, Jena, Germany). These images were then collected and analyzed by a Sensicam QE cooled digital camera system (Cooke Corporation, Germany) with MetaMorph/MetaFluor combination software package (Universal Imaging, West Chester, PA) and the results were assembled in Adobe Photoshop (Adobe, San Jose, CA).

### Analysis for the interaction between CchA and MidA by Yeast two-hybrid

The yeast two-hybrid analysis was performed using the Matchmaker Library Construction & Screening system (BD Clontech) [37]. For strain generation, a cDNA fragment corresponding to the cytosol C-terminus of CchA (1617–2110 amino acids) with SmaI-cch-5′ (TCCCCCGGGGCTGCTTTTTCCGGACGAGTT) and cch-BamHI-3′ (CGCGGATCCTTATGTCTCGTCCCTTGGTCG) was amplified and cloned into the pGADT7 vector, which contains the GAL4 DNA-AD and the LEU2 marker (BD Clontech). The full length cDNA of *midA* was placed in frame with the DNA-binding domain of GAL4 by PCR amplification with EcoRI-mid-5′ (CGGAATTCATGCAACGGCAAAACGC) and SmaI-mid-3′ (TCCCCCGGGCGCTAAAACACCATCACAAT) from the *A. nidulans* cDNA and subcloned into the pGBKT7 vector (Clontech, Palo Alto, CA), which contains the GAL4 DNA-BD and TRP1 marker (BD Clontech). *Saccharomyces cerevisiae* strain AH109 was used as the host for the two-hybrid interaction experiments. The histidine and adenine prototrophy and β-galactosidase assays were performed according to the clontech yeast protocols handbook (Clontech, Palo Alto, CA).

## Results

### CchA and MidA in *A. nidulans* are predicted homologs of Ca^2+^ voltage-gated and Ca^2+^-permeable, stretch-activated nonselective cation channels, respectively

**Figure 1:**
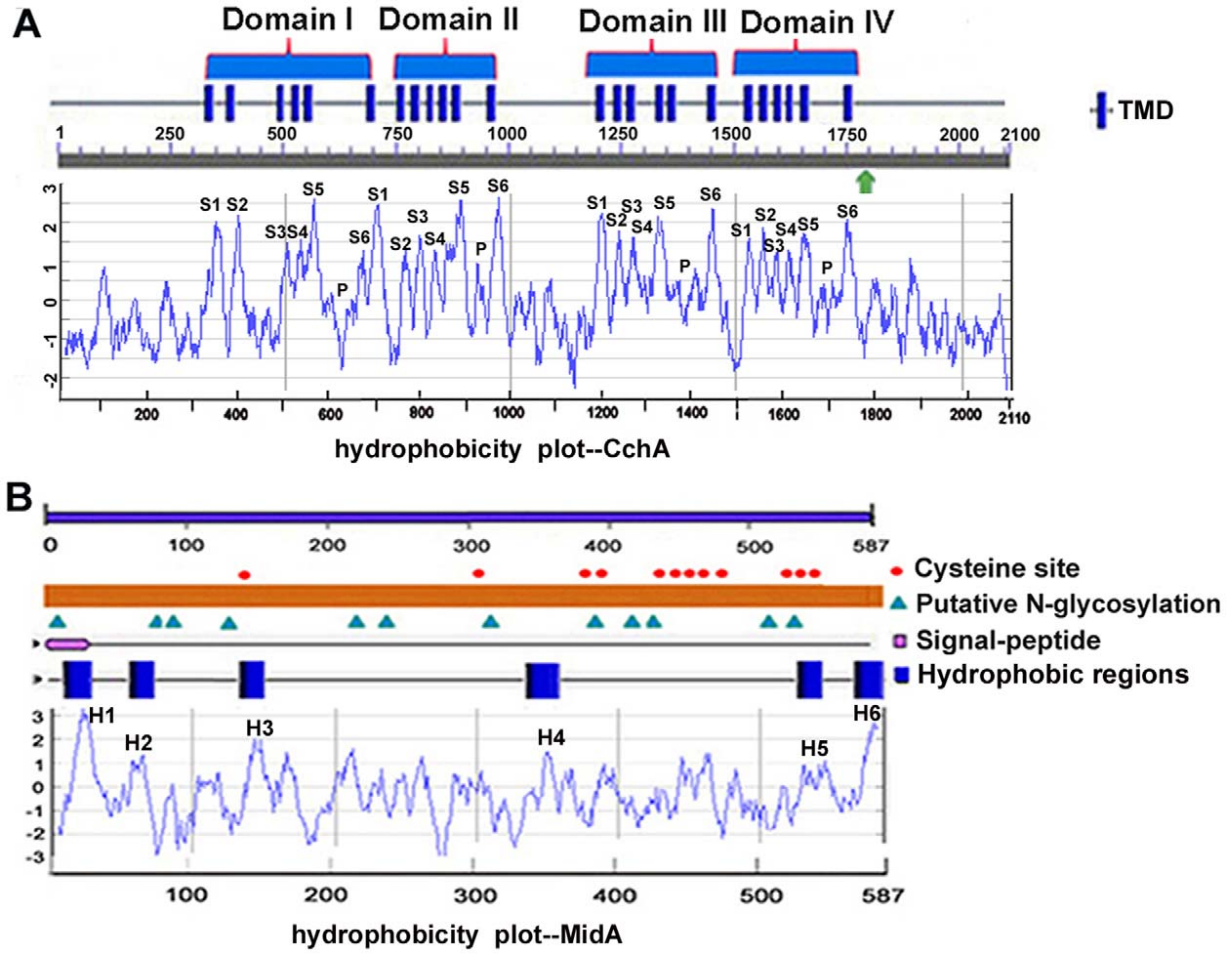
Identification of putative calcium channels-CchA and MidA in *A. nidulans*. (A) Predicted topology and Kyte-Doolittle hydropathy analysis profile of CchA in *A. nidulans*. CchA has four domains (I, II, III, and IV), and each consists of six membrane-spanning regions (S1 to S6). The pore loops (P) are located between S5 and S6 segments of each repeated domain. Cylinders indicate transmembrane domains (TMDs). Arrowhead indicates the position of EF-hand calcium-binding domain in CchA (1773–1808 aa). (B) Characteristics and Kyte-Doolittle hydropathy analysis profile of MidA. MidA has six hydrophobic regions (H1, H2, H3, H4, H5 and H6) predicted from the hydropathy profile, 12 putative N-glycosylation sites and 12 cysteine residues and a signal-peptide in N-terminal. Cylinders indicate transmembrane domains (TMDs). Position numbers of amino acid residues are indicated at the top of the figure.

Genomic comparative analyses by BLAST search in NCBI and CADRE show there are predicted plasma-membrane-located Ca^2+^ channel homologs of Cch1 and Mid1- CchA (GenBank accession no. AN1168.4 in NCBI and ANIA 01168 in CADRE) and MidA (GenBank accession no. AN8842.4 in NCBI and ANIA 08842 in CADRE) in *A. nidulans*. The predicted topology of CchA is similar to the overall topology of Ca^2+^ voltage-gated channels in higher eukaryotes. The *cchA* gene is 6,479 nucleotides long and contains three introns and four exons. It translates to a protein of 2,110 amino acids including an EF hand motif between the region of 1,773–1,808 amino acids and a signal peptide sequence between the regions of 1–29 amino acids. Hydropathy analysis revealed that the overall structural topology of CchA contained four repeated membrane domains (I, II, III, and IV), each consisting of six membrane-spanning regions (S1, S2, S3, S4, S5, and S6) included pore loop segments between S5 and S6, indicated by smaller hydrophobic indices (P) (Figure 1A). This predicted topology was consistent with the hydropathy profile determined by the algorithm of Kyte and Doolittle. It has been reported that most eukaryotic voltage-gated Ca^2+^ channels include four conserved glutamic acid residues in each of the four domains (I to IV), which facilitate high affinity divalent cation binding. However, CchA only has three of the glutamic acid residues in the pore regions of domains II, III, and IV (Figure S1), which is similar to that reported in *Cryptococcus neoformans* [14]. Thus, three glutamic acid residues are probably sufficient for promoting high-affinity calcium binding. The *midA* gene has a total length of 1,816 nucleotides, which comprises two exons and one intron translating to a protein including 587 amino-acid residues. As shown in Figure 1B, the predicted topology of MidA is similar to the overall topology of stretch-activated calcium channel homolog Mid1 in higher eukaryotes. With the schematic diagram and Kyte Doolittle hydropathy analysis, the full length of MidA includes six putative transmembrane domains (TMDs), 12 cysteine residues, and 12 putative N-glycosylation sites.

To understand the phylogenetic relationships of CchA and MidA homologs in filamentous fungi as compared to single-cell yeasts and other eukaryotic animals and plants, phylogenetic analyses were carried out and shown in Figure S2. Although the membrane-spanning regions in CchA homologs are relatively conserved, the sequence identities for whole sequences between fungi and animals or between fungi and plant are very low as shown in Figure S2A. Similarly, the identities of MidA homologs among filamentous fungi were relatively higher than those between filamentous fungi and single-cell yeast (Figure S2B). Interestingly, no MidA homolog was obtained from selected animals and plants.

### The construction and confirmation of the conditional and deletion strains of *midA* and *cchA*

**Figure 2:**
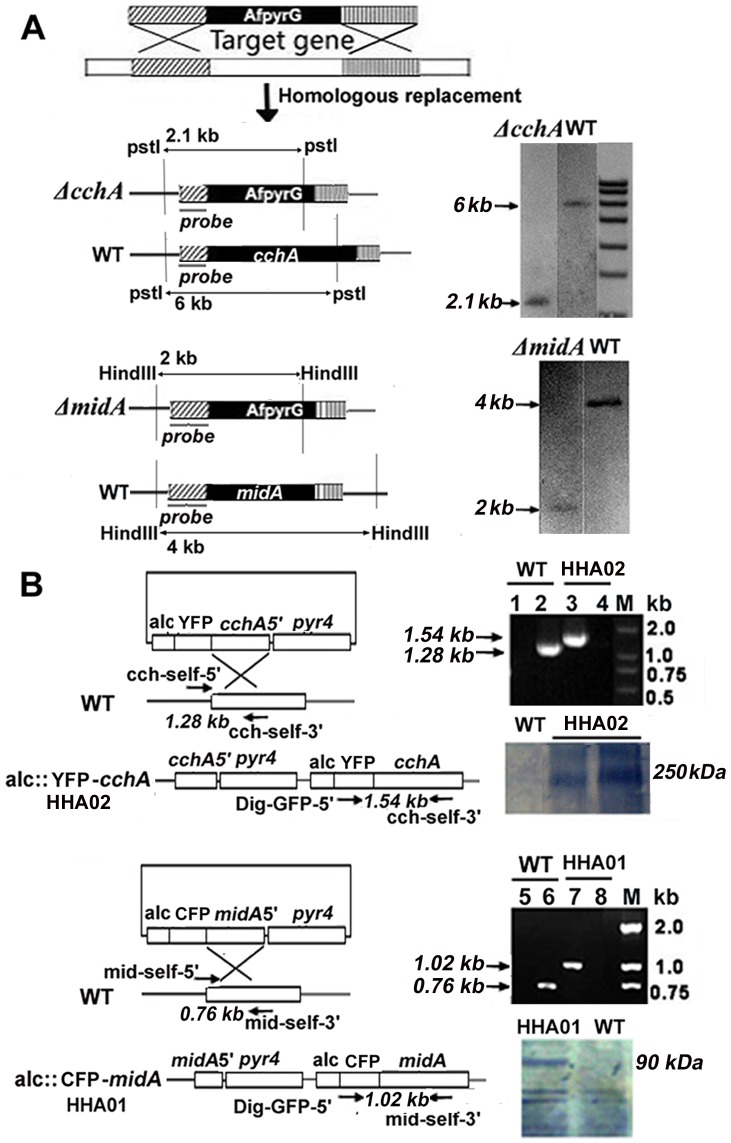
Constructions of the *cchA* and *midA* deletion and tagged strains. (A) Diagrams showing the strategy for generating *cchA* and *midA* deletion strains and the identification of homologous recombination by Southern blotting. The genomic DNA from the wild-type and the Δ*cchA* strains was digested with PstI; the DIG-labelled probe bound to a 6 kb and a 2.1 kb band in the wild-type and the Δ*cchA* strains respectively, indicating the replacement of *cchA* by *Afpyr*G. For Southern analysis of Δ*midA* strain, HindIII-digested genomic DNA of Δ*midA* mutant and wild type was blotted by a probe derived from retained regions of *midA*, showing the replacement of *midA* by *Afpyr*G. (B) Digrams showing the strategy for generating two condtional *alcA*(*p*)::YFP-*cchA* and *alc*A(*p*)::CFP-*midA* strains. Diagnostic PCR and Western blotting confirmed the homologous integration at the original *cchA* locus at the *alcA*(*p*)::YFP-*cchA* strain HHA02 and the original *midA* locus at the *alc*A(*p*)::CFP-*midA* strain HHA01. For lanes 1 and 3, PCR primers were Dig-GFP-5′ and cch-self-3′ to detect whether there was a homologous recombination to replace *cchA* with auxotrophy gene *AfpyrG* in the genome, and the expected size is 1.54 kb; for lanes 2 and 4, PCR primers were cch-self-5′ and cch-self-3′ to detect whether *cchA* still exists in the genome, and the expected size is 1.28 kb; for lanes 5 and 7, PCR primers were Dig-GFP-5′ and mid-self-3′ to detect whether there was a homologous recombination to replace *midA* with *AfpyrG* in the genome and the expected size is 1.02 kb; for lanes 6 and 8, PCR primers were mid-self-5′ and mid-self-3′ to detect whether *cchA* still exists in the genome, and the expected size is 0.76 kb. Western blotting analysis indicated the homologous integration at the original *cchA* locus in the *alcA*(*p*)::*cchA* strain and at the original *midA* locus at the *alc*A(*p*)::*midA* strain. A protein extract from wild-type strain was used as a negative control for the anti-GFP antibody.

To functionally characterize CchA and MidA in *A. nidulans*, two conditional strains (*alcA*(*p*):*:midA* strain HHA01 and *alcA*(*p*)::*cchA* strain HHA02) and four deletion mutants of *cchA* and *midA* (Δ*cchA* deletion strain WSA05, Δ*midA* deletion strain CJA08, C-terminus of *midA* deletion strain CJA09, and Δ*cchA*/Δ*midA* double deletion strain WSA06) were created by homologous integration according to the strategy illustrated in Figure 2 and Figure S3. Repeated single-spore isolations were performed to obtain stable homokaryotic deletion mutants. Diagnostic PCR analysis (Figure 2B) showed that both fusion alleles *alcA(p)*::*midA* and *alcA*(*p*)::*cchA* were located at the native gene loci where the inducible *alc*A(p) replaced the native promoters. Additionally, CFP and YFP were fused to N-terminus of MidA and CchA for Western analysis as described below. Single deletion mutants were also successfully constructed by homologous replacement of the *midA* and *cchA* ORFS with the *Afpyr*G gene (Figure 2A). Moreover, double mutants of *midA*/*cchA* were obtained by homologous replacement of *midA* with the gene *Anpyro*A, and transformed into the strain containing the *cchA* deletion background (Figure S3). Southern blot analysis confirmed in selected deletion mutants, the homologous integration of only one copy at the targeted locus *cchA* or *midA* genes (Figure 2A).

For the two conditional *alcA*(*p*)::*midA* and *alcA*(*p*)::*cchA* strains, Western blotting was performed to examine the expression of fusion proteins by GFP antibody, which can recognize all GFP variants. In denaturing lysates from the integrated HHA01, HHA02 and wild-type strains in the induced medium MMPGR, MidA-CFP and CchA-YFP were detected as the bands of approximately 90 and 250 kDa by the anti-GFP antibody. The predicted size of MidA and CchA GFP fusions, 63 and 238 kDa, was consistent with our results (Figure 2B). In comparison, there was no detectable band appeared in wild-type strain under the same condition. These data indicate that *alc*A(p)::CFP and *alc*A(p)::YFP had been integrated at the targeted locus *cchA* or *midA* genes in tagged strains and the anti-GFP antibody only was able to recognize the specific CFP or YFP signals.

### CchA and MidA are required for normal conidiation in minimal medium

**Figure 3:**
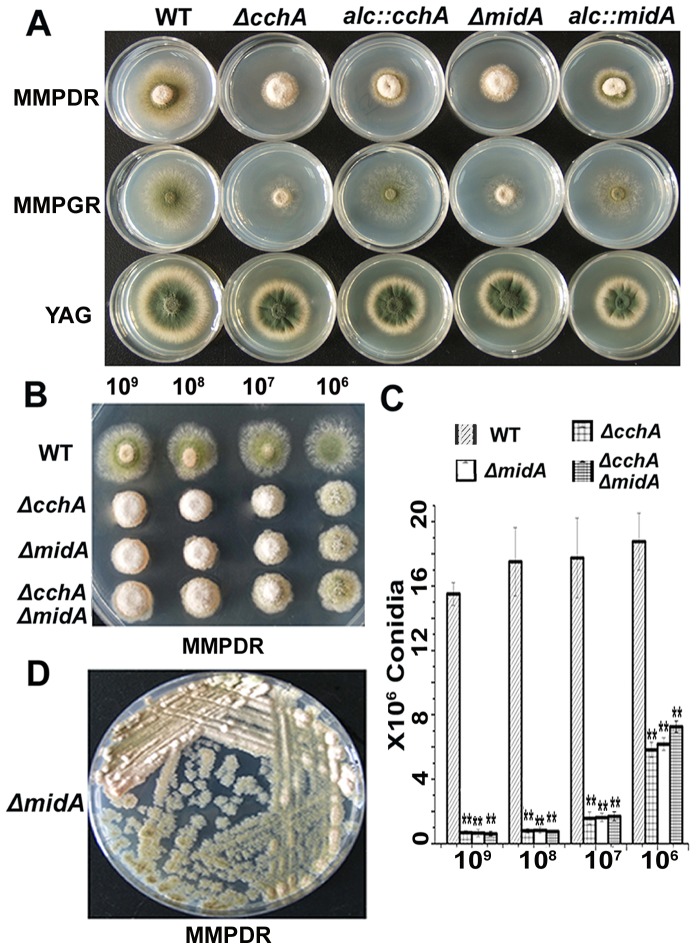
Depletion of CchA/MidA resulted in conidiation defects in an inoculum-size-dependent way. (A) Colony and conidiation phenotype comparison of wild-type, *alcA*(*p*)::*cchA*, *alcA*(*p*)::*midA*, Δ*cchA*, Δ*midA* strains. A total of 2 µl of 10^9^ conidia ml^−1^ were spotted on the induced (MMPGR), repressed (MMPDR and YAG) media respectively. (B) Conidiation phenotypes in wild-type and *cchA*/*midA* mutants at a series of 2 µl 10-fold dilutions derived from a starting suspension of 10^9^ conidia ml^−1^ on MMPDR. (C) The bar diagram showing the quantification of conidia for whole colony in a dilution-series of inoculum size as indicated. The value is means ± SD of three independent experiments. The significance was set at level **p<0.01 compared with the control group (WT). (D) A loopful of spores from Δ*mid*A strain CJA08 were streaked on MMPDR until to obtain single spore inoculum, then incubated at 37°C for 48 h, showing the conidiation defect of Δ*midA* was in an inoculum-size-dependent way.

To explore the effect of CchA/MidA on different growth media, we used the above-described strains to observe the colony morphology on supplemented minimal medium (MMPDR or MMPGR) or the rich medium YAG (Figure 3A). For conditional strains, the expression of *cchA* and *midA* was regulated by the carbon source: repression by glucose on YAG and MMPDR or non-repression by glycerol on MMPGR (Figure 3). As expected, the conditional strains expressing the *alc*(p)-GFP variant tagged version of CchA or MidA displayed an identical phenotype to the untagged wild-type strain when grown on MMPGR medium, demonstrating the functionality of the fusion protein. Notably, Δ*cchA*, Δ*midA* and conditional mutants on YAG and MMPDR media showed significantly smaller colony sizes, indicating that they had relatively low growth rates compared to that of the wild type. Moreover, all deletion mutants showed an aconidial phenotype in MM media. Consistently, as shown in Figure 3A, when down-regulated by the *alcA* promoter in the presence of glucose, both of *cchA* or *midA* conditional strains showed the similar and the consistent phenotypes with *cchA* or *midA* whole gene deletion strains, indicating the expression of *cchA* and *midA* were turned off successfully. However, these growth and development defects of the mutants were significantly suppressed when growing on YAG.

More interestingly, as shown in Figure 3B, the conidiation defect phenotype was induced in a density-dependent manner. From a series of 10-fold dilutions, the conidiation defect of the mutants was clearly reduced as the inoculum size decreased. Quantification of spores from these treatments confirmed these observations (Figure 3C), showing that at low densities, the *cchA*/*midA* double mutants produced approximately 40% of the spores produced by wild type compared to 4% at high densities. To continue confirm whether the conidiation defect phenotype in mutants can be suppressed by the low density of inoculation, we streaked a loopful of spores from *midA* deletion strain in one plate to show the colony phenotype with a series of inoculation dilutions until to single spore inoculum. Consistently, as shown in Figure 3D, the conidiation defect of *midA* was suppressed to some extent with the decreasing of inoculation.

**Figure 4:**
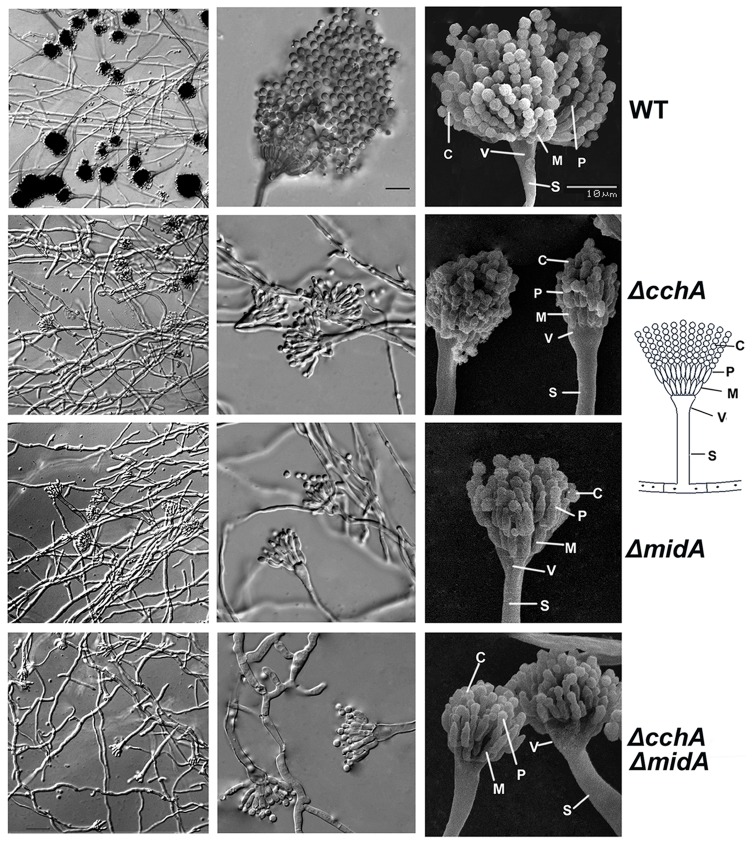
Comparison of microscopic phenotypes between wild-type and *cchA*/*midA* deletion mutants. The left and middle panels displayed the micrographs of Different Interference Constrast (DIC) for indicated strains. The *cchA*/*midA* mutants showed a sharp reduction in the number of conidiophores compared with wild-type strain. Characterization of conidiation defects in *cchA*/*midA* mutants indicated by the Scanning electron micrographs (SEM) in right panel. All stains were cultured on MMPDR at 37°C for 48 h. Abbreviation S, V, M, P and C labeled at right panel represent Stalk, Vesicle, Metula, Phialide and Conidiospores respectively. Bars, 10 µm.

To better understand these conidiation defects, a microcopy study was conducted. The results indicated that loss of either gene caused a sharp reduction in the number of conidiospores as well as abnormally shaped metulae and phialides (Figure 4). In the wild type, the vegetative hyphae developed into conidiophores with visible phialides and numerous conidia to produce the distinct ‘aspergillum’ in appearance. In comparison, deletion mutants formed fewer vesicles and some of distorted metulae and phialides. Most significantly, the mutants were unable to form chains of conidia.

### Conidiation defects of *cchA/midA* mutants can be rescued by extracellular Ca^2+^ in a calcineurin-dependent way but by osmotic stress in a calcineurin-independent way

**Figure 5:**
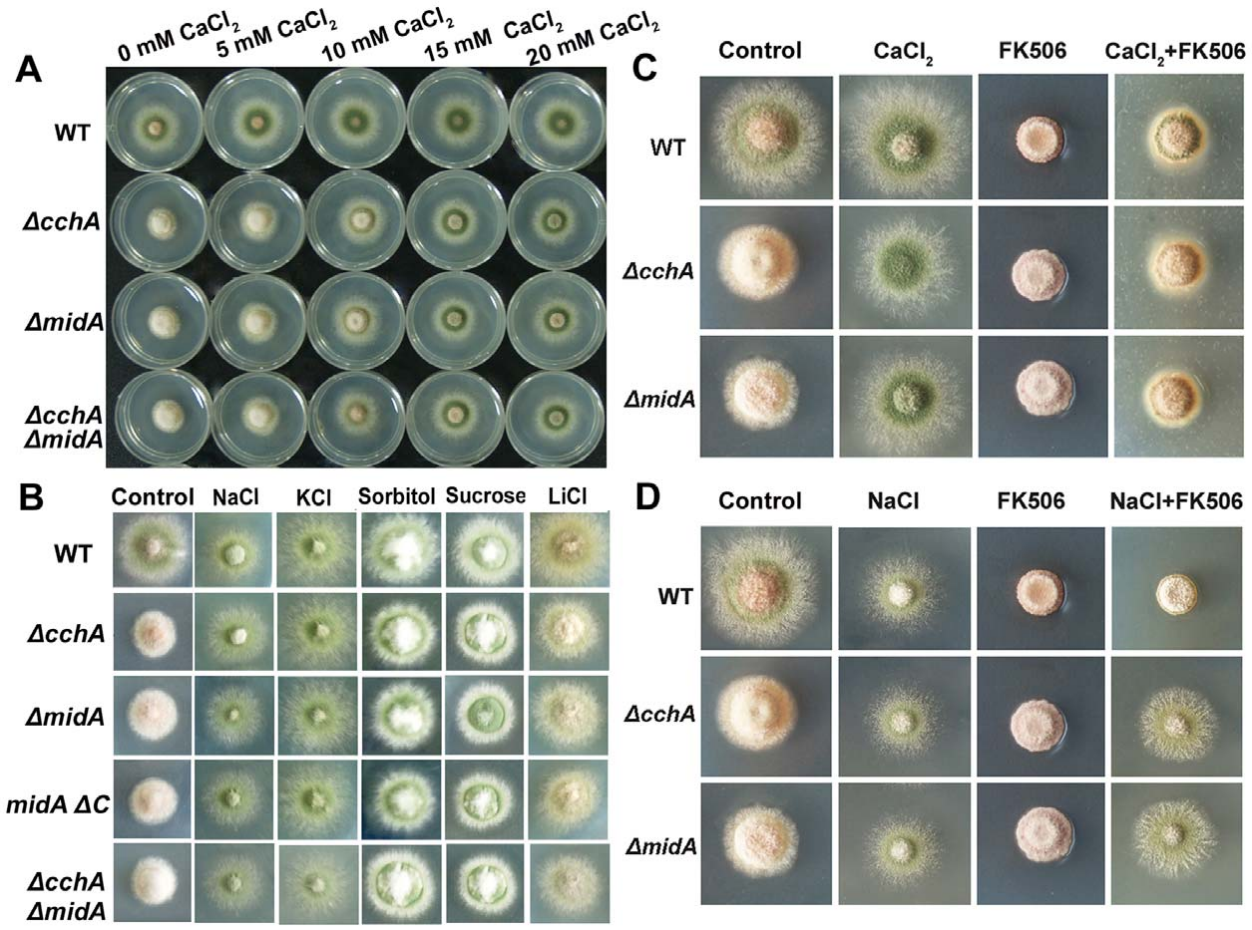
The conidiation defects of *cchA*/*midA* mutants can be rescued by extra-cellular Ca^2+^ and osmotic or cation stress. (A) Conidiation defects of *cchA*/*midA* deletion mutants can be rescued by extra-cellular Ca^2+^. The indicated strains were inoculated onto MMPDR with 2 µl of 10^9^ conidia ml^−1^ supplemented with different doses of CaCl_2_ as indicated. (B) Conidiation defects of *cchA*/*midA* deletion mutants can be rescued by osmotic stress or ion stress. 2 µl of 10^9^ conidia ml^−1^ were spotted onto MMPDR supplemented with 800 mM NaCl, 600 mM KCl, 1 M Sorbitol, 1 M Sucrose, 200 mM LiCl respectively. (C) Conidiation defects of *cchA*/*midA* deletion mutants can be rescued by extra-cellular Ca^2+^ in calcineurin-dependent way. 2 µl of 10^9^ conidia ml^−1^ were spotted onto MMPDR supplemented with 40 mM CaCl_2_, 1 µM FK506 and 40 mM CaCl_2_ plus 1 µM FK506 respectively. (D) Conidiation defects of *cchA*/*midA* deletion mutants can be rescued by osmotic stress in a calcineurin-indenpendent way. 2 µl of 10^9^ conidia ml^−1^ were spotted onto MMPDR supplemented with 800 mM NaCl, 1 µM FK506 and 800 mM NaCl plus 1 µM FK506 respectively. All plates were incubated at 37°C for 48 h.

Our results above suggested that conidiation in *A. nidulans* depends on the function of CchA/MidA when grown in low-calcium environments. Because CchA and MidA are thought to encode subunits of the high-affinity Ca^2+^ channels, the most likely reason for the defects in mutants would be net calcium transport. To address this hypothesis, we inoculated mutant spores on the MMPDR media supplemented by adding different concentrations of CaCl_2_ (Figure 5A). As expected, Ca^2+^ substantially improved the sporulation of the mutants on minimal media in a dose-dependent manner. Previous findings from ours and others labs have indicated that the net calcium transport can be induced by osmotic stress [5], [7]. Therefore, we also grew the Δ*cchA* and Δ*midA* mutants on several stressors to see if osmotic or ion stress could restore wild type growth. As shown in Figure 5B, the conidiation defect phenotype could be suppressed to some extent by these stresses indicating osmotic stress or ion stress could partly mimic the function of extracellular Ca^2+^, even in a low calcium minimal media. Based on the ideas described in previously published data, calcineurin is probably a central player in the calcium signaling pathway, we asked whether calcineurin, as an indicator of a high-affinity calcium influx system, was required for rescue of conidiation in the mutants. When the calcineurin inhibitor-FK506 was added to MMPDR media, neither wild-type or deletion mutants were able to sporulate (Figure 5C, 5D). Moreover, in wild type, adding extracellular Ca^2+^ or osmotic stress (0.8 M NaCl) was not able to substantially improve the FK506 conidiation defect. Most interestingly, in *cchA* or *midA* deletion strains, 0.8 M NaCl but not extracellular Ca^2+^ was able to rescue the FK506 defect (Figure 5C, 5D). These data suggest that conidiation defects in mutants can be rescued by either extra-cellular Ca^2+^ in a calcineurin-dependent way or osmotic stress in a calcineurin-independent way.

### CchA and MidA deletions lead to the loss of the apical dominant axis of growth and are more resistant to cell wall damaging agents

**Figure 6:**
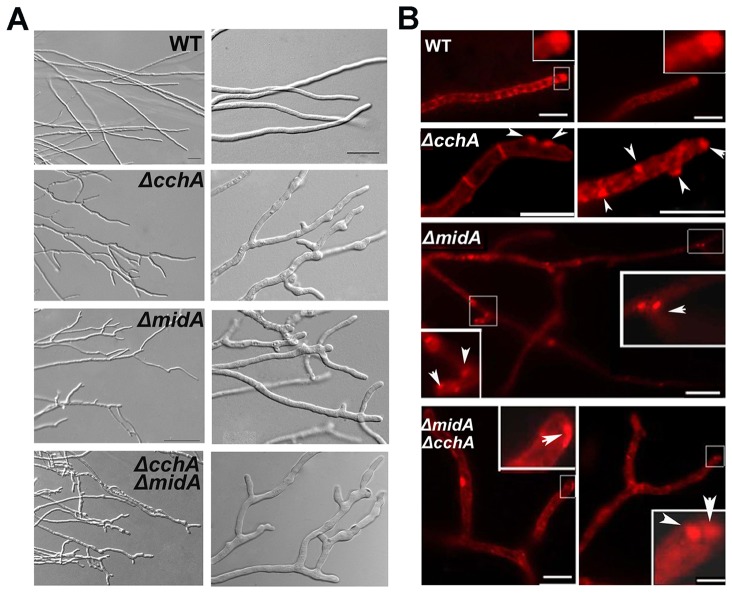
CchA/MidA deletion leads to the loss of apical dominant axis of growth. (A) Hyphal DIC morphology in wild-type and *cchA*/*midA* mutants cultured on MMPDR for 16 h at 37°C. Compared with the wild type, Δ*cchA*/Δ*midA* single or double mutants were significantly impaired in the formation of a single axis of hyphal polarity. (B) The fluorescence distribution of hyphal cells stained by FM4-64 in wild-type and mutants. Arrowheads indicate FM4-64 abnormally distributed in *cchA*/*midA* deletion mutants. Bars, 10 µm.

The colony phenotype of the mutants (Figure 3) suggested aberrancy in polar growth. This phenotype raised the question of whether the apical dominance of hyphae was defective in these deletion mutants. To examine this hypothesis, we microscopically assessed growth of these strains cultured on MMPDR. Figure 6A shows that Δ*cchA*/Δ*midA* single or double mutants were significantly impaired in the formation of the single axis of hyphal polarity, resulting in the hypha branched early along with an abnormally wide and a bulbous growth pattern. This was in contrast to wild-type, which had organized, parallel, and defined hyphal filaments. In addition, we found that the mutant hyphae branched repeatedly to yield multiple polarity axes such that there was no single dominance; by contrast, wild-type had apical dominance as a result of suppression of secondary polarity axes in the general vicinity of a growing hyphal tip under the same culture conditions. Moreover, the phenotype of hyphal polarity defect is not exacerbated by the presence of the double deletion.

Previous studies have shown that the Spitzenkörper (SPZ), located at hyphal tips, plays a key role in enforcing apical dominance and the spatial regulation of polar growth [46]. Therefore, localization of the SPZ results in the characteristic shape of the hyphal tip. We thought it possible that the polarity defects in the deletion mutants are related to the abnormal accumulation or position of SPZs. To address this question, the localization of SPZs, as found by staining with FM4-64 which is a membrane-selective fluorescent vital dye reported as markers of endocytosis in the live cells, was observed. With the increasing of incubation time of FM4-64 with the cell, they entered the cell primarily by endocytic vesicles invaginated from the plasma membrane. Based on our observation, after adding stain, firstly, the plasma membrane was immediately stained, outlining the typical shape of the hyphal cell or germlings. Later on, the plasma membranes at the septum site were often seen, showing the septum outline. Next, numerous submicrometre-sized fluorescent organelles including the Spitzenkörper (SPZ) were seen. Figure 6B shows that most of the hyphal cells had FM4-64 stained spots at the hyphal apex in the wild-type. In contrast, the distribution of FM4-64 in null mutants showed a remarkably abnormal pattern, in which the non-uniform staining of FM4-64 in hyphae apparently allowed the hypha to direct the growth randomly. These data suggest that CchA and MidA play important roles in the positioning of SPZ at the hyphal apex.

**Figure 7:**
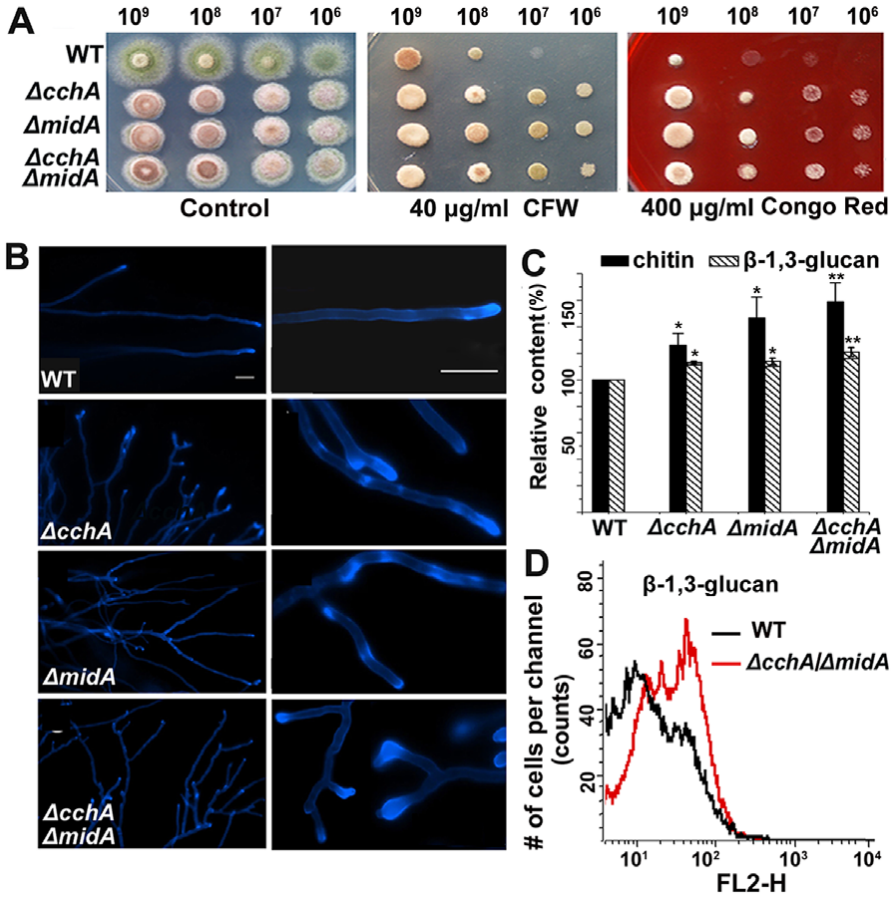
Deletion of *cchA*/*midA* affects cell wall components and resistance to cell wall–damaging agents. (A) Comparison of resistance towards cell wall–perturbing agents between *cchA*/*midA* deletion mutants and wild-type strain. A series of 2 µl 10-fold dilutions derived from a starting suspension of 10^9^ conidia ml^−1^ as indicated were spotted onto MMPDR supplemented with 40 µg ml^−1^ Calcofluor White and 400 µg ml^−1^ Congo Red respectively and incubated at 37°C for 2 days. (B) The visualization of chitin stained with Calcofluor White (CFW). Spores were incubated onto coverslips covered with MMPDR, incubated at 37°C for 16 h. The mycelium of strains were stained with 5 µg ml^−1^ CFW for 5 min. (C) Comparison of relative chitin and β-1, 3-glucan contents between wild-type and mutants. The experiment was performed in biological triplicate for statistical analysis. The significance was set at level *p<0.05 and **p<0.01 between mutants and wild-type strain. (D) Monitoring of the β-1, 3-glucan level of wild-type and Δ*cchA/*Δ*midA* strains using β-1, 3-glucan antibody by flow cytometry.

Because *mid*1 deletion is known to be sensitive to cell wall stress in other systems [17], plate assays with standard cell wall stressors, Calcofluor White (CFW: 40 µg ml^−1^) and Congo Red (CR: 400 µg ml^−1^), were performed. As shown in Figure 7, all strains including wild-type and mutants showed a significant decrease in growth rate under cell wall stress conditions. However, the growth in single and double mutants was unexpectedly less impaired than that in the wild type, suggesting that *cchA* and *midA* are more resistant to cell wall damaging agents than is the wild-type strain.

Because Chitin and β-1, 3-glucan represent the main structural components of the fungal cell wall, we suspected that the components of cell walls in mutants had possibly been changed. Therefore, we first assessed the patterns of chitin deposition by fluorescence microscopy using Calcofluor White. The result showed many cell wall depositions and bright chitin spots irregularly distributed, possibly reflecting a difference in cell wall architecture. To further analyze the components of the cell wall in mutants, we next examined the content of chitin and β-1, 3-glucan. As shown in Figure 7C and Table S2, the chitin content increased significantly in mutants, indicating that the deletion of CchA and MidA caused a significant change in the polysaccharide content of the cell wall. In comparison, the β-1, 3-glucan content in mutants also showed the statistically significant increased but the difference was much less than that of chitin. Consistently, flow cytometry analysis labeled by β-1, 3-glucan antibody revealed that the mutant strains had the differential rearrangement of surface carbohydrate epitopes compared with the wild type (Figure 7D).

### Cytosol C-terminus of CchA interacts with MidA in Y2H

**Figure 8:**
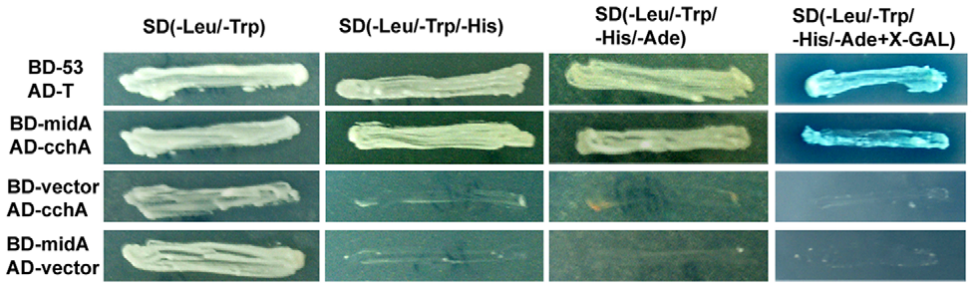
Physical interaction between CchA and MidA revealed by yeast two-hybrid system. Full length cDNA of *midA* placed in frame with DNA-bi nding domain of GAL4 in the pGBKT7 vector was referred as BD-*midA* while a truncated cytosol C-termini fragment of *cchA* inserted into the pGADT7 vector was named as AD-*cchA*. Protein–protein interaction was detected by the growth of transformed yeast strains AH109 on Y2H screen media with high stringency (SD/-Ade/-His/-Leu/-Trp/X-α-gal) and low stringency (SD/-Leu/-Trp), respectively. pGADT7-T labeled AD-T and pGBKT7-p53 labeled by BD-p53 were used as the positive controls for the interaction.

Based on the fact that deletion of CchA results in the same phenotype as seen for MidA, it is quite possible that CchA and MidA function together in *A. nidulans*. To further assess the relationship between CchA and MidA, yeast two-hybrid system was used to provide the direct evidence of an interaction between CchA and MidA. Because the predicted topology of CchA was similar to the overall topology of Ca^2+^ voltage-gated channels in higher eukaryotes, CchA may function as an alpha subunit of the calcium channel. As described in the information on the Ca^2+^ voltage-gated channels, the predicted interaction region of the alpha-subunit with the beta-subunit belonged to the cytosol C-terminus of CchA. According to this notion, we constructed a C-terminal fusion of CchA with the Gal4-DNA binding domain and a full length of MidA with the GAL4-activation domain. We did so because the GAL4-DNA activation domain can activate transcription, but require a DNA-binding domain to recognize the UAS-DNA sequence to activate transcription. Consequently, the yeasts transfected by pGBKT7-CchA-C and pGADKT7-MidA showed a robust growth in high stringency media (Figure 8), indicating that the reporter genes (histidine, adenine prototroph, and beta-galactosidase activity) could be activated. None of the yeast cells transfected by single pGBKT7-CchA-C or single pGADKT7-MidA grew under high stringency media, suggesting that none of the bait and prey plasmids had detectable auto-activation. In comparison, the positive colonies of pGADT7-T with pGBKT7-p53 had robust growth under the same cultural condition. These results demonstrate that the cytosol C-terminus of CchA physically interacts with MidA in Y2H assays.

## Discussion

Up to date, high-affinity and low-affinity calcium uptake systems (HACS and LACS) have been reported in a variety of fungi, especially in yeast [7], [8], [15]. It has been verified that HACS play important roles in facilitating calcium influx at low concentrations and in stress responses, including mating stress, osmotic stress and other plasma membrane perturbations in fungi [7], [47]. Moreover, calcium channels Cch1 and Mid1 have been implicated as the high-affinity calcium uptake system [14], [15], [16], [17]. However, calcium channels have not been identified previously in the *Aspergilli*. In this study, our study is the first to verify the functional characterization of *A. nidulans* putative calcium channels by conditional and null deletion mutants. Our data consistently indicate that CchA and MidA not only are functionally required under low-calcium environmental condition—which is consistent with their homologs of Cch1 and Mid1 in yeast—but they also have unique and complex roles in regulating conidiation development, hyphal polarity establishment, and cell wall composition in low-calcium environments in the filamentous fungus *A. nidulans*.

### CchA and MidA may play important roles in hyphal polarity maintenance and cell wall structure

Previous studies have reported strong evidence that SpitzenkÖper (SPZ) (rich in microfilaments, ribosomes, secretory vesicles, and chitosomes) at the hyphal tips plays a key role in enforcing apical dominance and the spatial regulation of polar growth [45], [46]. Therefore, localization of SPZ results in the characteristic shape of the hyphal tip. Our data suggest that the polarity defects in deletion mutants are related to the abnormal accumulation position of SPZ. Thus, the predicted calcium channel CchA and MidA play important roles in the accumulation position of SPZ at the hyphal apex for maintaining the apical dominant axis of growth and ensuring the hyphal growth direction. Moreover, the cell wall of yeasts and filamentous fungi consists of a fibrillar network of polysaccharides that protects the cell from external stress and maintains cell shape and flexibility [46], [48], [49], [50]. This process seems to be disturbed when CchA and MidA are deleted because Δ*cchA*/Δ*midA* single or double mutants were significantly impaired in the formation of a single axis of hyphal polarity, resulting in a randomly crossed filament pattern as well as abnormal hyphal shape. We believe that the unbalanced accumulation of cell wall polysaccharides and loss of the dominance of the apical SPZ in growing hyphae of *cchA*, *midA* or double mutants may explain these phenotypes [46]. Furthermore, the fact that Δ*cchA*/Δ*midA* single or double mutants are more resistant to cell wall damaging agents than is the wild-type strain suggests that CchA and MidA are probably involved in the cell wall integrity pathway. Indeed, as shown in Figure 7, chitin content is significantly increased in mutants, indicating that the deletion of CchA or MidA may have resulted in a significant change in the polysaccharide content of the cell wall. In comparison, previous studies revealed that the increased chitin synthesis in *S. cerevisiae* is possibly related to resistance to cell wall damaging agents [51]. Thus, elevating chitin synthesis in *cchA* and *midA* may be a general complementary cellular strategy for the defected cell wall.

### Involvement of CchA and MidA in conidiation in a density-dependent manner

In *A. nidulans*, conidiation initiates with the foot cell, which develops a conidiophore; then, at the tip of the conidiophore, the stalk swells to form a multinucleated vesicle, from which metulae are produced. The metulae bud to create uninucleated sporogenic cells called phialides. The asexual spores or conidia are produced by repeated mitotic divisions of the phialide nucleus so that each mature phialides will produce about 100 asexual spores in wild type *A. nidulans* under the proper conditions [23], [52]. We found that all of the colonies of deletion mutants or conditional strains in repressed media showed a fluffy, near aconidial phenotype compared to that of the wild-type, which exhibited robust developed conidiophores in minimal media under the same condition. Furthermore, osmotic stress could partly mimic the function of extracellular Ca^2+^, even in a low calcium minimal media. It suggests that in the absence of CchA/MidA, an alternative Ca^2+^ influx pathway might exist, and the pathway might have a relatively low Ca^2+^ affinity; additionally, it might function on a higher calcium media. These data suggest that the putative calcium channel CchA and MidA plays an important role in regulating the developmental process of conidiation in *A. nidulans*. Interesting, the repression of conidiation in the mutants was density dependent as lower densities of cells yielded more normal conidial numbers. Conidiation has been associated with quorum-like density dependence in the fungus *A. flavus* [53], [54], in that case associated with a balance between asexual (high densities) and sexual (low densities) development. The quorum molecules associated with this system are oxygenated fatty acids called oxylipins. Recently subsets of these oxylipins have been found to stimulate conidiation in *A. nidulans* [55]. Possibly the CchA/MidA calcium channel is required for availability or reception of quorum-like signals – such as oxylipins - important for conidiation.

### The concerted action of CchA/MidA with calcineurin during the conidiation

Based on the ideas described in previously published data, calcineurin is probably a central player in the calcium signaling pathway [7]. Recent investigations have revealed that calcineurin, a Ca^2+^/calmodulin-activated protein phosphatase, has an essential role in morphogenesis, virulence, and antifungal drug action in two life-threatening pathogenic fungi *C. albicans* and *C. neoformans* [21]. Thus, calcineurin inhibitors FK506, CsA or other analogs hold promise as novel candidates for antifungal drugs [56], [57], [58], [59]. Similarly, in this paper, we found that FK506 was able to inhibit conidiation either under normal or osmotic stress conditions. This suggests that calcineurin could possibly be a good target of antifungal drugs in fungi [60], [61]. However, our findings in Figure 5 indicate that, in the absence of CchA or MidA, calcineurin inhibitor FK506 is not able to block conidiation under NaCl stress conditions, resulting in a significant rescue of the conidiation defect in the mutants in this treatment. In the absence of CchA/MidA, conidiation defects can be remarkably rescued by osmotic stress in a calcineurin-independent way apparently blocked a FK506 response pathway in the fungus. These data imply that under certain stress conditions, defects in the calcium channel enable a bypass of the requirement of calcineurin for conidiation. Further studies are needed to elaborate the relationship between calcineurin and plasma-membrane-located Ca^2+^ channels and the mechanism behind these stress responses. Nevertheless, our findings in this paper enlighten a potentially viable and completely unexplored avenue to control conidiation in *Aspergilli* by the high affinity Ca^2+^ channel. Thus, understanding the mechanism of HACS function will be of great help in finding novel drug therapies for fungal infections.

## Supporting Information

Figure S1 **Sequence alignments and analyses of CchA.** Multiple sequence alignments of the loop region in CchA homologs in *H. sapiens*, *M. musculus*, *S. cerevisiae*, *C. albicans*, *C. neoformans and A. nidulans*. CchA shows similarity to *H. sapiens* Cav1.2 channel in the pore region. Three of the four glutamic acid residues (E) presented in the pore regions of domains II, III, and IV of the L-type Ca^2+^ are conserved in CchA. The locations of acidic residues forming an acidic ring motif in Cav1.2 channels are indicated by black solid circle. The overall motif (EEEE, NEEE, NENE) formed by all four domains at this locus are indicated to the right of the Domain IV alignment for each channel homologue.
(TIF)

Figure S2 **Phylogenetic homology analysis of CchA and MidA homologs in selected organisms.** (A) Phylogram shows the homology distance of CchA homolog with full-length sequences. The graphs are constructed using neighbor-joining method from amino acid sequences of following CchA homologs: *A. nidulans* (AF393474_1), *A. fumigatus* (XP_752476.1), *A. clavatus* (XP_001269155.1), *A. terreus* (XP_001210398.1), *A. oryzae* (BAE64105.1), *A. niger* (XP_001392456.1), *P. chrysogenum* (XP_002559315.1), *N. crassa* (XP_963732.2), *C. neoformans* (XP_570175.1), *C. albicans* (AAN86029.1), *S. cerevisiae* (EEU05742.1), *D. melanogaster* (NP_727772.2), *H. sapiens* (NP_001122311.1), *M. musculus* (NP_001077085.1), *A. thaliana* (AAD11598.1). (B) Phylogram shows the homology distance of MidA homolog with full-length sequences. The amino acid sequences of MidA homologues are followed: *A. nidulans* (XP_682111.1), *A. fumigatus* (XP_754048.1), *A. flavus* (XP_002382957.1), *A. oryzae* (XP_003189076.1), *A. niger* (XP_001398435.1), *P. marneffei* (XP_002148196.1), *C. posadasii* (XP_003069581.1), *G. zeae* (XP_387594.1), *N. crassa* (XP_961018.2), *C. purpurea* (CAU66903.1), *C. albicans* (XP_710952.1), *S. cerevisiae* (EGA81234.1), *C. neoformans* (XP_569171.1).
(TIF)

Figure S3 **Diagrams showing the strategy for generating Δ** ***midA*** **- trancated-C terminus and** ***cchA*** **/** ***midA*** **double deletion strains.** The C-terminus coding sequence of *midA* was replaced with the *pyrG* and homologous recombination was confirmed by diagnostic PCR. The entire coding sequence of *midA* was replaced with the *pyroA* in the Δ*cchA* strain and homologous recombination was confirmed by diagnostic PCR. For lanes 1 and 3, PCR primers were mid-self-5′ and diag-pyrG-3′ to detect whether there was a homologous recombination to replace *midA* with auxotrophy gene *AfpyrG* in the genome, and the expected size is 1.52 kb; for lanes 2 and 4, PCR primers were mid-self-5′ and mid-p6 and the expected size is 2.2 kb in WT and 3.0 kb in Δ*midA*-trancated-C terminus strain; for lanes 5 and 7, PCR primers were mid-self-5′ and mid-self-3′ to detect whether *midA* still exists in the Δ*cchA*, and the expected size is 0.76 kb; for lanes 6 and 8, PCR primers were mid-p1 and pyro-3′ to detect whether there was a homologous recombination to replace *midA* with auxotrophy gene *AnpyroA* in Δ*cchA*, and the expected size is 2.4 kb. For lane 1, 2, 5, 6, the template was WT genomic DNA. For lane 3 and 4, lane 7 and 8, genomic DNA of Δ*mid*A-trancated-C terminus and double mutant was used as PCR template, respectively.
(TIF)

Table S1 **Primers used in this study.**
(DOCX)

Table S2 **Cell wall composition of** ***A.nidulans*** **wild type and mutant strains.**
(DOCX)
