# Supplementary figures and images for: Putative Calcium Channels CchA and MidA Play the Important Roles in Conidiation, Hyphal Polarity and Cell Wall Components in Aspergillus nidulans
Source: PLoS One. 2012 Oct 12;7(10):e46564. doi: 10.1371/journal.pone.0046564 (PMC3470553; doi:10.1371/journal.pone.0046564)

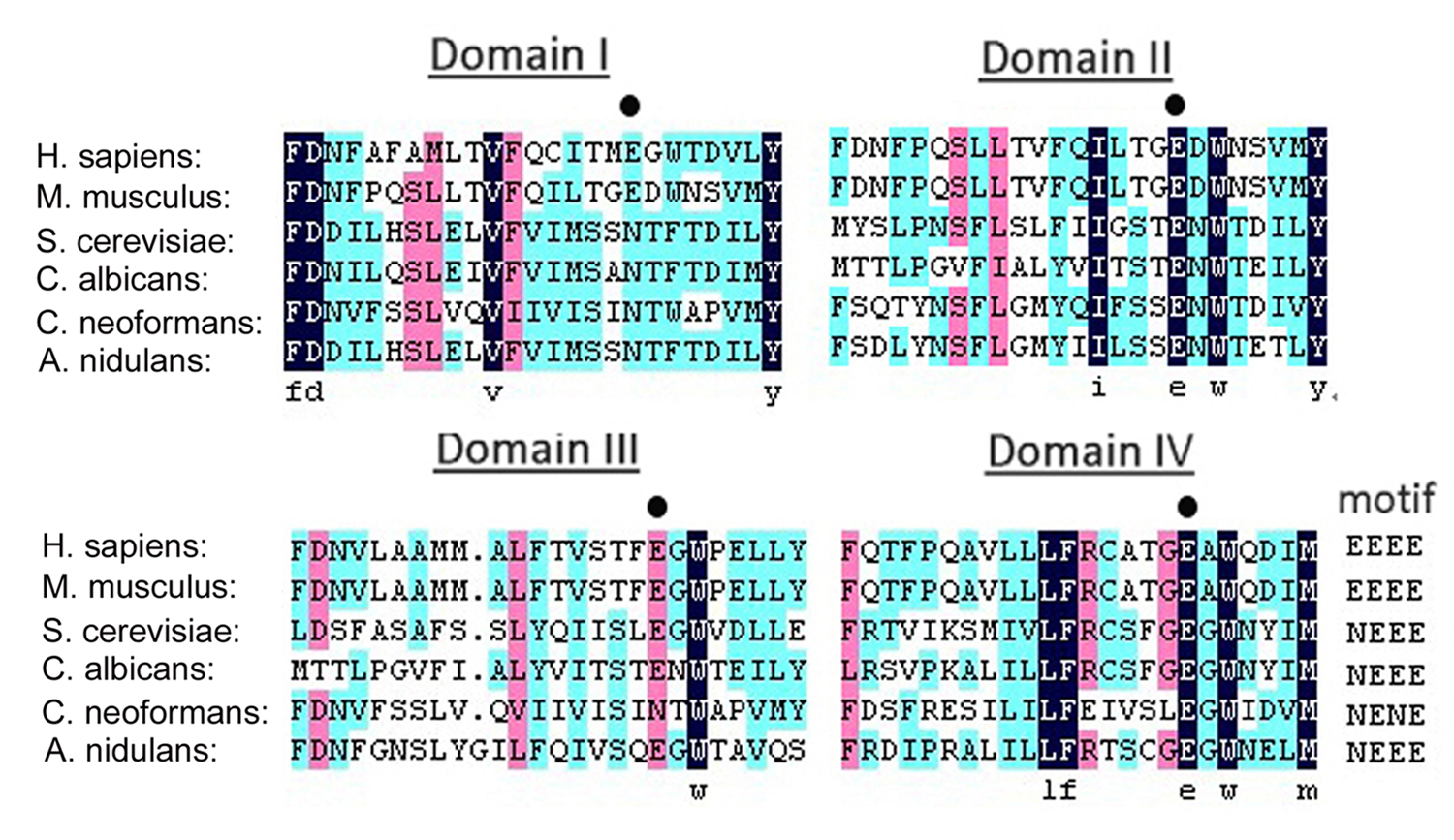

Supplement: Figure S1 — Sequence alignments and analyses of CchA. Multiple sequence alignments of the loop region in CchA homologs in H. sapiens, M. musculus, S. cerevisiae, C. albicans, C. neoformans and A. nidulans. CchA shows similarity to H. sapiens Cav1.2 channel in the pore region. Three of the four glutamic acid residues (E) presented in the pore regions of domains II, III, and IV of the L-type Ca2+ are conserved in CchA. The locations of acidic residues forming an acidic ring motif in Cav1.2 channels are indicated by black solid circle. The overall motif (EEEE, NEEE, NENE) formed by all four domains at this locus are indicated to the right of the Domain IV alignment for each channel homologue. (TIF) [file pone.0046564.s001.tif]

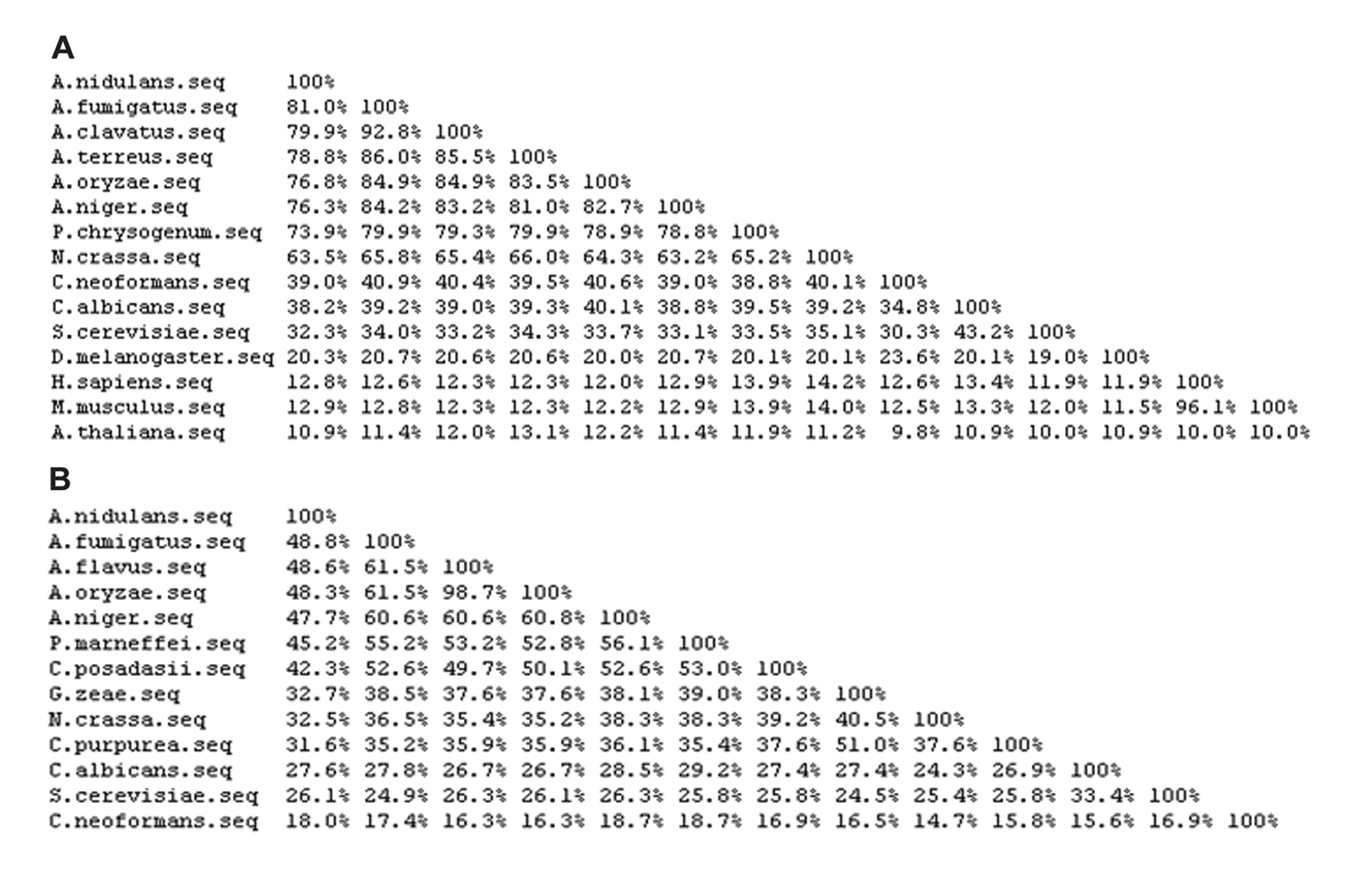

Supplement: Figure S2 — Phylogenetic homology analysis of CchA and MidA homologs in selected organisms. (A) Phylogram shows the homology distance of CchA homolog with full-length sequences. The graphs are constructed using neighbor-joining method from amino acid sequences of following CchA homologs: A. nidulans (AF393474_1), A. fumigatus (XP_752476.1), A. clavatus (XP_001269155.1), A. terreus (XP_001210398.1), A. oryzae (BAE64105.1), A. niger (XP_001392456.1), P. chrysogenum (XP_002559315.1), N. crassa (XP_963732.2), C. neoformans (XP_570175.1), C. albicans (AAN86029.1), S. cerevisiae (EEU05742.1), D. melanogaster (NP_727772.2), H. sapiens (NP_001122311.1), M. musculus (NP_001077085.1), A. thaliana (AAD11598.1). (B) Phylogram shows the homology distance of MidA homolog with full-length sequences. The amino acid sequences of MidA homologues are followed: A. nidulans (XP_682111.1), A. fumigatus (XP_754048.1), A. flavus (XP_002382957.1), A. oryzae (XP_003189076.1), A. niger (XP_001398435.1), P. marneffei (XP_002148196.1), C. posadasii (XP_003069581.1), G. zeae (XP_387594.1), N. crassa (XP_961018.2), C. purpurea (CAU66903.1), C. albicans (XP_710952.1), S. cerevisiae (EGA81234.1), C. neoformans (XP_569171.1). (TIF) [file pone.0046564.s002.tif]

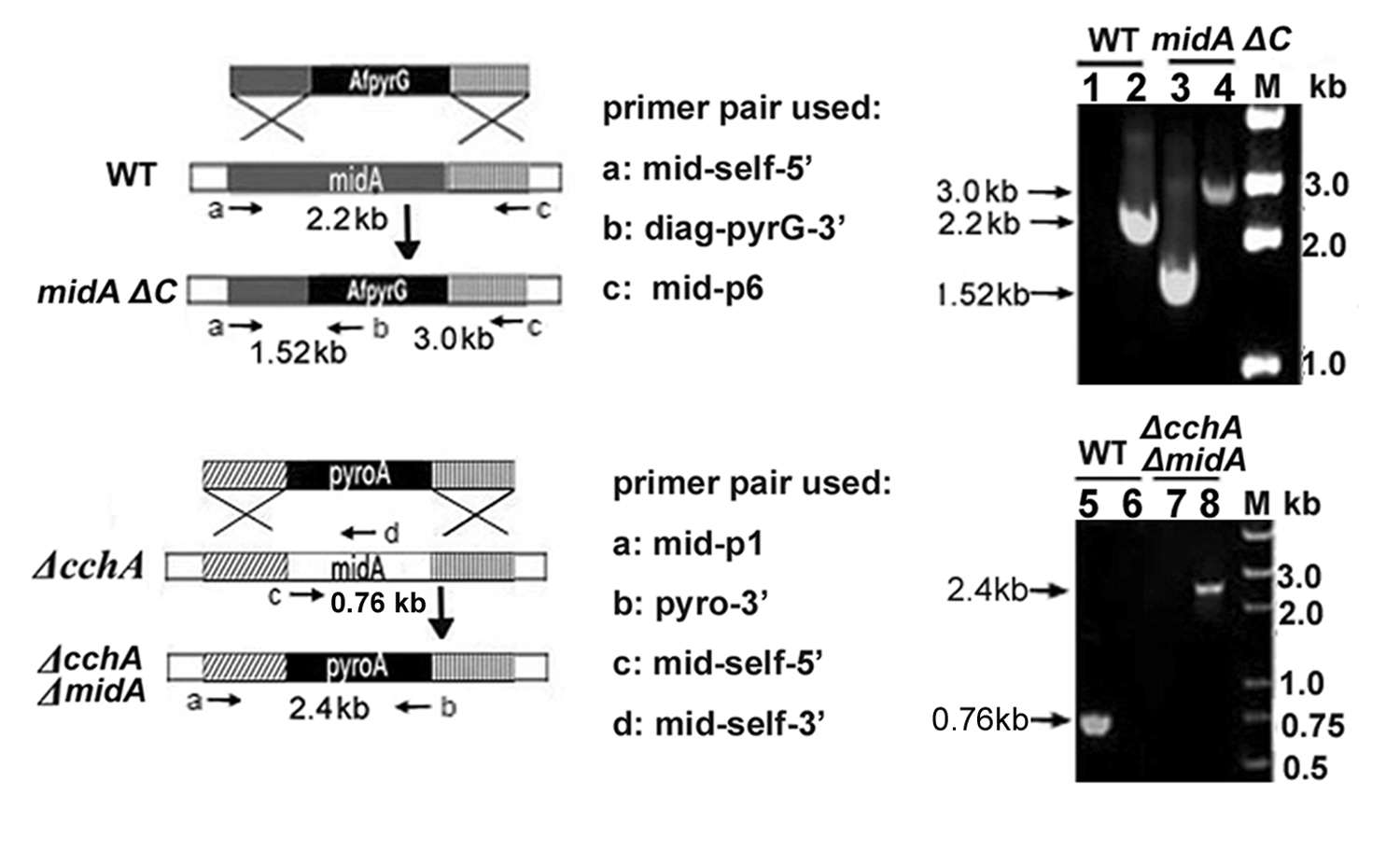

Supplement: Figure S3 — Diagrams showing the strategy for generating Δ midA - trancated-C terminus and cchA / midA double deletion strains. The C-terminus coding sequence of midA was replaced with the pyrG and homologous recombination was confirmed by diagnostic PCR. The entire coding sequence of midA was replaced with the pyroA in the ΔcchA strain and homologous recombination was confirmed by diagnostic PCR. For lanes 1 and 3, PCR primers were mid-self-5′ and diag-pyrG-3′ to detect whether there was a homologous recombination to replace midA with auxotrophy gene AfpyrG in the genome, and the expected size is 1.52 kb; for lanes 2 and 4, PCR primers were mid-self-5′ and mid-p6 and the expected size is 2.2 kb in WT and 3.0 kb in ΔmidA-trancated-C terminus strain; for lanes 5 and 7, PCR primers were mid-self-5′ and mid-self-3′ to detect whether midA still exists in the ΔcchA, and the expected size is 0.76 kb; for lanes 6 and 8, PCR primers were mid-p1 and pyro-3′ to detect whether there was a homologous recombination to replace midA with auxotrophy gene AnpyroA in ΔcchA, and the expected size is 2.4 kb. For lane 1, 2, 5, 6, the template was WT genomic DNA. For lane 3 and 4, lane 7 and 8, genomic DNA of ΔmidA-trancated-C terminus and double mutant was used as PCR template, respectively. (TIF) [file pone.0046564.s003.tif]
