# Supplementary material for: Putative Calcium Channels CchA and MidA Play the Important Roles in Conidiation, Hyphal Polarity and Cell Wall Components in Aspergillus nidulans
Source: PLoS One. 2012 Oct 12;7(10):e46564. doi: 10.1371/journal.pone.0046564 (PMC3470553; doi:10.1371/journal.pone.0046564)
Supplement: Table S1 — Primers used in this study. (DOCX) [file pone.0046564.s004.docx]

**Table S1.** Primers used in this study.

| **Primer** | **DNA sequence (5'-3')** |
| --- | --- |
| SmaI-cch-5' | TCCCCCGGGGCTGCTTTTTCCGGACGAGTT |
| cch-BamH I-3' | CGCGGATCCTTATGTCTCGTCCCTTGGTCG |
| EcoR I-mid-5' | CGGAATTCATGCAACGGCAAAACGC |
| SmaI-mid-3' | TCCCCCGGGCGCTAAAACACCATCACAAT |
| cchA-5' | TTATGCGGCCGCTGGCGTCAAATAGCCATGAC |
| cchA-3' | TTGGTCTAGACCATTCCGATTGCGCTGATTC |
| mid-5' | ACTTGGCGGCCGCTGCAACGGCAAAAC |
| mid-3' | TACACCCGGGTACACCTCAGATGTAG |
| cchA–p1 | CTGCACAAGAAGTTCTAGACGC |
| cchA–p2 | CGCAATTTACGCAAAGTCTCGC |
| cchA–p3 | CTCTAGATGCATGCTCGAGCGGGTGGGCAGAATTGATTGAC |
| cchA–p4 | CAGTGCCTCCTCTCAGACAGATAAAGCGCCGCTGAAGTGAG |
| cchA–p5 | TAGACTGGAGTTTTGTGCCGAC |
| cchA–p6 | CGAGTTGGGTTGTGCCTCTAGT |
| midA–p1 | GACCGGCCGTTCGCAAAATAC |
| midA–p2 | GCATTGGTTCATGTTGACTTG |
| midA–p3 | CTCTAGATGCATGCTCGAGCGAGGTCAAGTGCTCCGATC |
| midA–p4 | CAGTGCCTCCTCTCAGACAGCTCATGTCCAAGCGGAAAG |
| midA–p5 | GTACTATGACCAAAGGTCAATG |
| midA–p6 | GCGTGTAGTTGTCCTTATTTG |
| L-flank-5'(truncated) | ATGCAACGGCAAAACGCGC |
| L-flank-3'(truncated) | CTCTAGATGCATGCTCGAGCGTACACCTCAGATGTAGCAC |
| mid-p2(truncated) | GCACAGTCTCGCTTTGGTG |
| pyrG5' | GCTCGAGCATGCATCTAGAG |
| pyrG3' | CTGTCTGAGAGGAGGCACTG |
| Dig-GFP-5' | GACACCCTCGTCAACAGGATCG |
| Cch-self | TGTCTGCATTCGGCTAGTC |
| Cch-post | CTGGCCGGTGAATATCTAAGAG |
| Cch-up | TGTGATTAGCGACAGCAAGAGT |
| Cch-down | GACGCTCCTTCTGAGAAACTTG |
| Mid-self-5' | CATGCGTCAACAGTGTTGC |
| Mid-post-3' | GAATAGGATCCAGAGAAGTTC |
| Diag-pyrG | TAGGGACCGAGACCTGTATC |
| pyro-5' | TTGGCGGGTAAGTCAGATAATAG |
| pyro-3' | CTGACTTGAC GCTTTCTCTTGG |
| mid-L-pyro-p3 | GGAGCAAAGCAGGAGAATAGGTCAAGTGCTCCGATC |
| mid-R-pyro-p4 | TCTTGGCTCTATCGTATTCTTCTCATGTCCAAGCGGAAAG |
