# Supplementary material for: Putative Calcium Channels CchA and MidA Play the Important Roles in Conidiation, Hyphal Polarity and Cell Wall Components in Aspergillus nidulans
Source: PLoS One. 2012 Oct 12;7(10):e46564. doi: 10.1371/journal.pone.0046564 (PMC3470553; doi:10.1371/journal.pone.0046564)
Supplement: Table S2 — Cell wall composition of A.nidulans wild type and mutant strains. (DOCX) [file pone.0046564.s005.docx]

**Table S2.** Cell wall composition of *A.nidulans* wild type and mutant strains.

| Strain | Chitin  [μg (mg mycelial tissue)^-1^] (%) | β-1,3-glucan  [fluorescence unit] (%^a^) |
| --- | --- | --- |
| WT | 27.05 + 3.44 (100) | 222.72 + 10.61 (100) |
| △midA | 31.05 + 1.54* (115) | 249.45 + 9.58* (112) |
| △cchA | 34.28 + 1.75* (127) | 249.22 + 5.97* (112) |
| △midA/△cchA | 38.49 + 1.32** (142) | 263.67 + 4.87** (118) |

Data are presented as mean + SD for at least three experiments. The significance was set at level *p<0.05 and **p<0.01 compared with wild type strain control values.

^a^ relative fluorescence ratio = value of mean fluorescence reading in wild type strain/ value of mean fluorescence readings in respective mutant. The value of fluorescence reading in wild type strain was set at 100%.
